# Transplantation of Expanded Fetal Intestinal Progenitors Contributes to Colon Regeneration after Injury

**DOI:** 10.1016/j.stem.2013.09.015

**Published:** 2013-12-05

**Authors:** Robert P. Fordham, Shiro Yui, Nicholas R.F. Hannan, Christoffer Soendergaard, Alison Madgwick, Pawel J. Schweiger, Ole H. Nielsen, Ludovic Vallier, Roger A. Pedersen, Tetsuya Nakamura, Mamoru Watanabe, Kim B. Jensen

**Affiliations:** 1 Wellcome Trust & Medical Research Council Cambridge Stem Cell Institute, University of Cambridge, Cambridge, CB2 1QR, UK; 2 Anne McLaren Laboratory for Regenerative Medicine, Department of Surgery, University of Cambridge, Cambridge, CB2 0SZ, UK; 3 BRIC: Biotech Research and Innovation Centre, University of Copenhagen, DK-2200 Copenhagen N, Denmark; 4 Department of Gastroenterology and Hepatology, Tokyo Medical and Dental University, Bunkyo-ku, Tokyo, 113-8519, Japan; 5 Department of Gastroenterology, Medical Section, Herlev Hospital, Faculty of Health and Medical Sciences, University of Copenhagen, DK-2730 Herlev, Denmark

## Abstract

Regeneration and homeostasis in the adult intestinal epithelium is driven by proliferative resident stem cells, whose functional properties during organismal development are largely unknown. Here, we show that human and mouse fetal intestine contains proliferative, immature progenitors, which can be expanded in vitro as Fetal Enterospheres (FEnS). A highly similar progenitor population can be established during intestinal differentiation of human induced pluripotent stem cells. Established cultures of mouse fetal intestinal progenitors express lower levels of Lgr5 than mature progenitors and propagate in the presence of the Wnt antagonist Dkk1, and new cultures can be induced to form mature intestinal organoids by exposure to Wnt3a. Following transplantation in a colonic injury model, FEnS contribute to regeneration of colonic epithelium by forming epithelial crypt-like structures expressing region-specific differentiation markers. This work provides insight into mechanisms underlying development of the mammalian intestine and points to future opportunities for patient-specific regeneration of the digestive tract.

## Highlights

• The small intestine has a transient population of highly proliferative progenitors
• Maturation of fetal intestinal progenitors requires Wnt stimulation
• Lgr5+ fetal cells are in a permissive state for transitioning into the adult state
• Fetal intestinal progenitors differentiate in vivo upon transplantation

## Graphical Abstract

**Figure:**
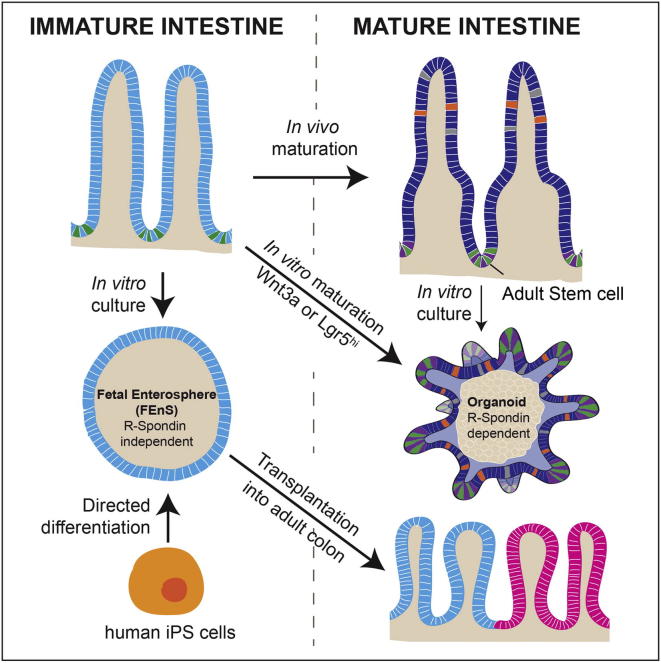

## Teaser

Fetal intestine contains expandable immature progenitors, which can also be derived from human pluripotent stem cells, that contribute to regeneration of injured colon following transplantation.

## Introduction

Fertilization of the oocyte initiates a series of events that, following gastrulation, leads to organ formation in the developing fetus. During this process, pluripotent stem cells progressively lose potential as the early embryo is patterned along its axes and organ structures are specified. Tissue-specific programs subsequently direct the formation and maturation of adult organs, which are maintained throughout life by stem cells with tissue-restricted lineage potential. It remains unclear whether transitory stem cell states exist in the embryo, responsible for tissue maturation, or whether maturation is achieved via adult tissue-specific stem cells in the fetal tissue. Understanding the process of tissue maturation in vivo has implications for the directed differentiation of pluripotent cells into functionally mature tissue types (Zorn and Wells, 2009).

The intestinal epithelium is continuously replenished by resident stem cells. The mature mammalian small intestine is a tube-like structure with an inner epithelial lining facing the lumen. This layer is organized into differentiated villi protruding into the lumen and proliferative crypt compartments invaginated into the underlying mesenchyme. Intestinal Stem Cells (ISCs) reside at the crypt base and give rise to all the differentiated cell types (Barker et al., 2007, 2012). Development of the small intestine follows a specific pattern. Villus formation in humans begins around the ninth week of gestation and embryonic day 15 (E15) in mouse. In the human, crypt formation occurs before birth, whereas in the mouse this happens during the first 2 postnatal weeks (Montgomery et al., 1999; Spence et al., 2011a). Beyond these morphological rearrangements, the mechanisms of initial intestinal lineage differentiation and functional maturation are less well characterized. Despite temporal differences in the ontogeny of the small intestine between human and mouse, the overall process of development is identical, making the mouse an accessible model to interrogate the process of human intestinal maturation.

Our understanding of the mature intestine has been accelerated by the establishment of culture conditions for long-term maintenance of adult mouse and human intestinal epithelium in vitro (Jung et al., 2011; Sato et al., 2009, 2011a). In this system, single ISCs or dissociated crypt fragments are embedded in Matrigel where they exhibit self-organization into “mini-guts.” Here we describe the identification of proliferative progenitors captured in the human fetal intestine and during intestinal differentiation of human induced pluripotent stem cells (hiPSCs). This is recapitulated in murine tissues, where fetal progenitors can transition spontaneously and by Wnt induction into an adult state. Finally, we present evidence that fetal progenitors can contribute to the regeneration of adult colonic epithelium in vivo, as proof of principle that developmentally immature cells have clinical potential.

## Results

### Fetal Human Intestinal Epithelium Can Be Propagated Long-Term In Vitro as Fetal Enterospheres

**Figure 1:**
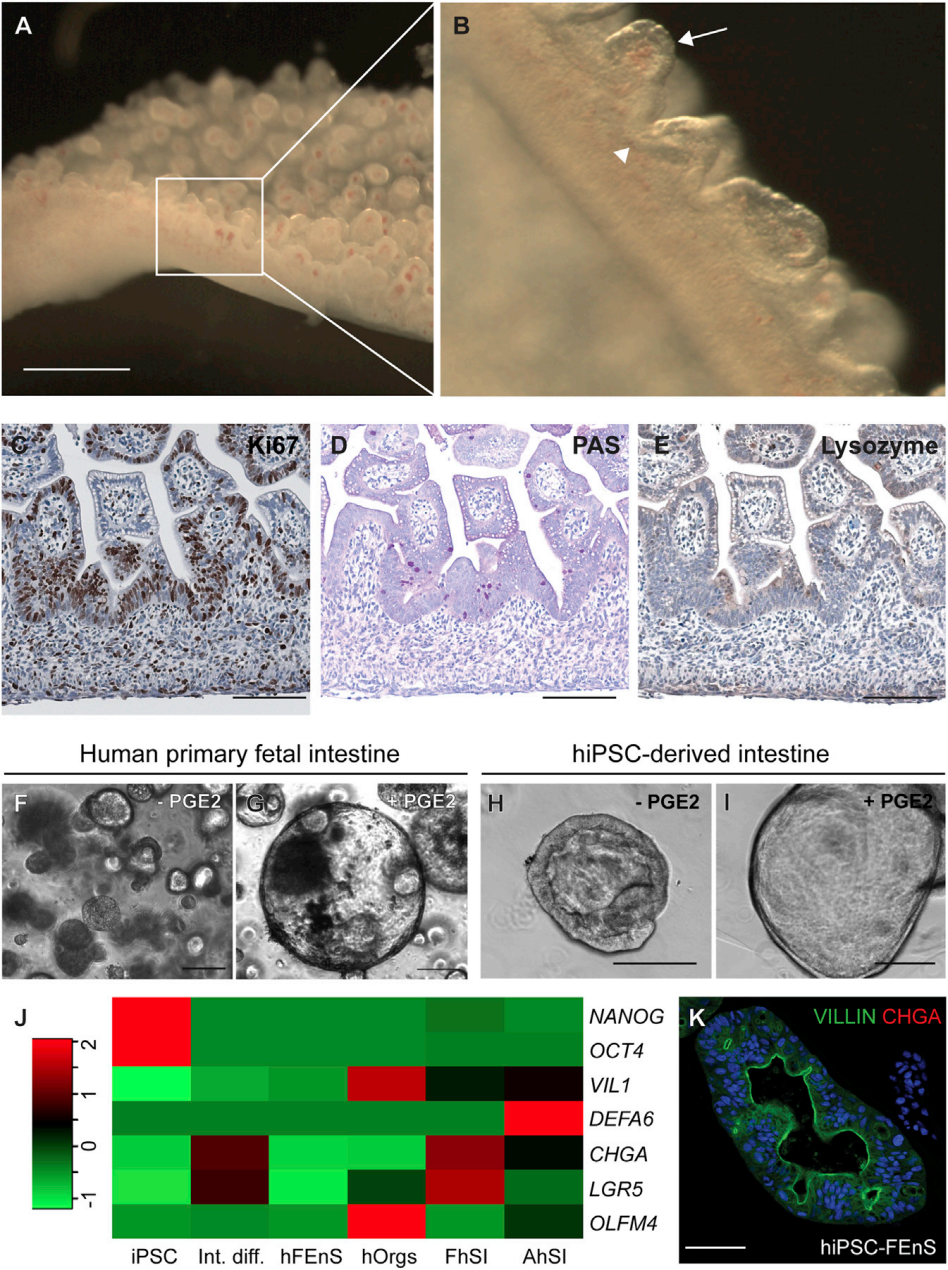
Derivation of Immature Intestinal Progenitors from Human Fetal and Pluripotent Cells (A) Whole mount of human gestational week 10 small intestine. (B) Higher magnification of villi (arrow) and intervillus regions (arrowhead) in (A). (C–E) Immunohistochemistry analysis for Ki67 (C), PAS staining (D), and Lysozyme (E) in week 10 human small intestine. (F and G) Spheroid cultures from week 10 human small intestinal epithelium, grown with (G) and without (F) prostaglandin E2 (PGE2) (2.5 μM). (H and I) Intestinal tissue derived from directed differentiation of human induced pluripotent stem cells (hiPSCs), cultured with (I) and without (H) PGE2. (J) Relative expression levels of intestinal lineage markers in material from undifferentiated human induced pluripotent stem cells (hiPSC), iPSC-derived intestine (Int. diff.), human primary fetal enterospheres (hFEnS), human adult organoids (hOrgs), primary fetal human small intestine (FhSI), and primary adult human small intestine (AhSI). Red and green colors reflect increased and decreased deviation from the mean, respectively. (K) Detection of VILLIN (green) and CHGA (red) in hiPSC-FEnS. The scale bars represent 2 mm in (A) and 100 μm in (C)–(E) and (K). See also Figure S1 and Table S1.

Previous studies have described the establishment of organoid cultures from mature human gut epithelium (Jung et al., 2011; Sato et al., 2011a). To investigate the in vitro potential of immature gut epithelium, we analyzed human fetal intestinal tissue around gestational week 10. At this stage, crypts have not formed and the human intestine consists of a series of undulating villi, with proliferation localized primarily to the intervillus regions (Figures 1A–1C). Here a subset of cells is weakly positive for Periodic Acid Schiff’s (PAS), though they do not have the mature morphology of goblet cells and there are no detectable Lysozyme^+ve^ Paneth cells (Figures 1D and 1E). The reduced level of secretory differentiation was confirmed at the transcriptional level (Figure 1J).

Fetal human intestinal tissue at around gestational week 10 was dissected and dissociated epithelial fragments were seeded in Matrigel. The conditions used for propagation of adult murine organoids (EGF, Noggin, and R-spondin1 [ENR]) caused the growth of small granular spheres that could not be maintained long-term without the addition of prostaglandin-E2 (PGE2) (Figures 1F and 1G). We term these human Fetal Enterospheres (hFEnS). hFEnS are highly proliferative and can be passaged repeatedly by mechanical dissociation for over 2 months with no spontaneous transition into budding organoids during this time.

### Intestinal Tissue from Human Pluripotent Cells Has Fetal Characteristics

Human induced pluripotent stem cells (hiPSCs) can be differentiated into intestinal epithelium (Spence et al., 2011b). We set out to determine whether hiPSC-derived intestinal tissue transitions through a fetal state. Using a chemically defined protocol, PSCs were directed toward definitive endoderm (DE) and further patterned into posterior DE (Hannan et al., 2013). Raised aggregates of cells forming from the sheet of posteriorized endoderm were transferred as small clumps to Matrigel (Figure S1A available online). Again PGE2 facilitated the formation of larger cystic epithelial spheroids, morphologically analogous to primary hFEnS (Figures 1H and 1I). These structures were maintained for over 2 months, through repeated passaging. In both cases PGE2 provides a pro-proliferative signal that drives the growth of spherical structures. hiPSC-FEnS also require low levels of Wnt3a to support growth, suggesting that although morphologically alike, they possess slightly different properties. Expression analysis verifies the immature nature of human FEnS and hiPSC-FEnS when compared to human adult organoids as well as fetal and adult intestine (Figures 1J and S1B). iPSC-derived FEnS had Villin present at the apical cell membrane in the spherical structure, and its immature nature is further supported by the lack of secretory Chromogranin-A^+ve^ cells (Figure 1K).

### Establishment of FEnS from Immature Mouse Intestine

**Figure 2:**
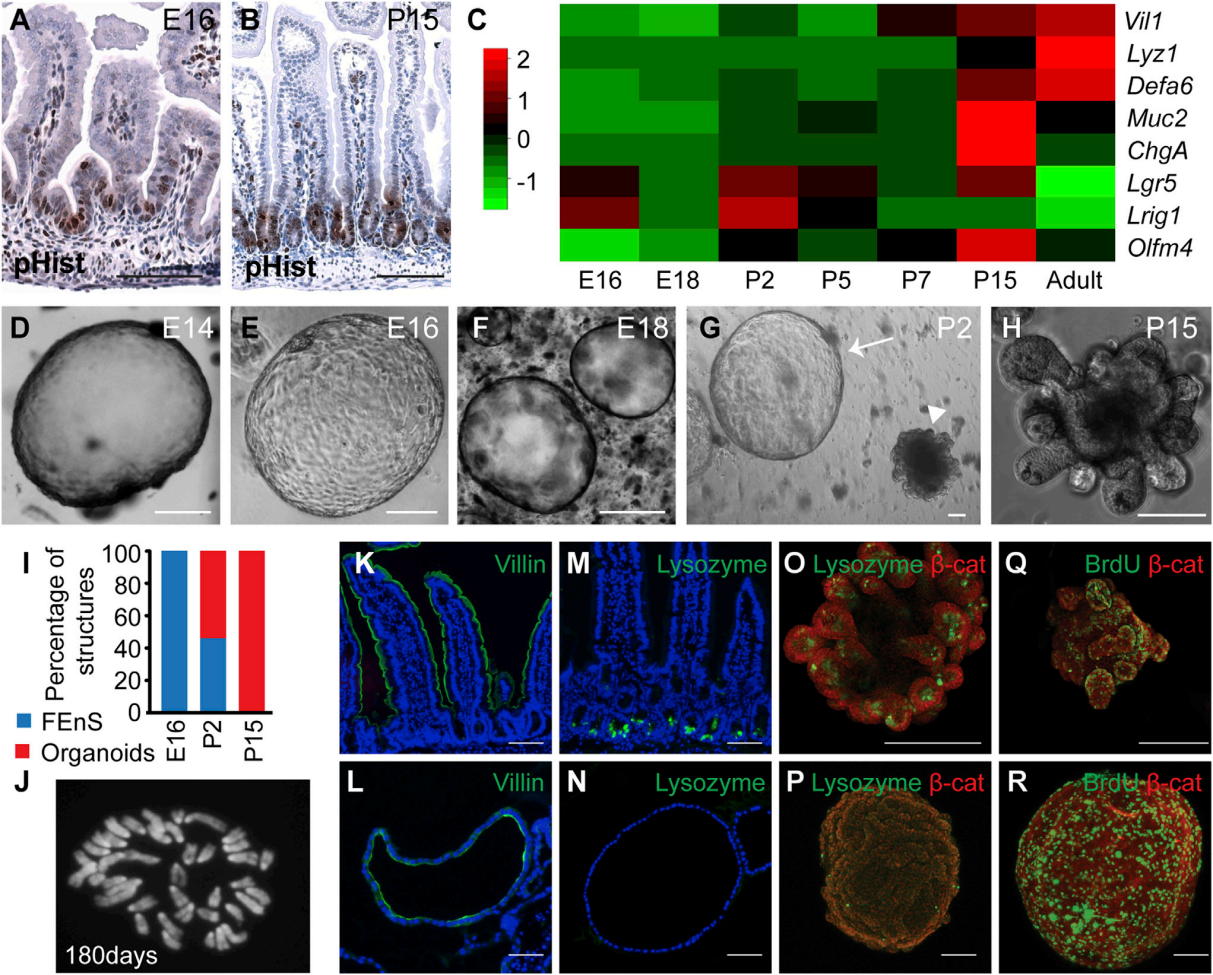
Establishment of mFEnS from Immature Mouse Intestine (A and B) Immunohistochemistry analysis for Phospho-Histone-H3 (pHist) on sections of small intestine from E16 mice (A) and P15 mice (B). (C) Relative expression levels of intestinal lineage markers in tissue isolated from proximal murine intestine at increasing developmental age from E16 to adult. Red and green colors reflect increased and decreased deviation from the mean, respectively. (D–H) Representative images of in vitro structures derived from E14 to P15. The arrow and arrowhead in (G) indicate an FEnS and an organoid, respectively. (I) Relative proportions of FEnS and organoids present after 2 weeks from E16, P2, and P15 tissues. (J) Metaphase spread of a cell at day 180 shows a normal karyotype (n = 15). (K and L) Detection of apical villin expression (green) in adult small intestine (K) and mFEnS (L). (M–P) Lysozyme expression in adult small intestine (M), cross sections of mFEnS (N), and whole-mount organoids and mFEnS (O and P). (Q and R) BrdU incorporation analysis in whole mounts of organoids and FEnS (green). β-catenin (red) is used as a counterstain. The scale bars represent 100 μm. E, embryonic day; P, postnatal day; adult, >3 weeks postnatal. See also Figures S2 and S3.

We reasoned that development of the mouse intestine would provide an accessible model system to interrogate intestinal maturation more closely. The mouse intestine at embryonic day 16 (E16) resembles the human intestine at around 10 gestational weeks with high proliferation in the intervillus regions and scattered immature goblet cells (Figures 2A, S2A, S2B, S2E, and S2F). By postnatal week 2, mature crypts are forming (Figures 2B, S2C, and S2D) and mature Lysozyme^+ve^ Paneth cells can now be detected in the proliferative zones (Figures S2G and S2H). The appearance of secretory cells is also evident by expression analysis during the course of intestinal development (Figure 2C).

To investigate whether fetal murine intestine contains equivalent FEnS progenitors, we seeded epithelial cells from the proximal half of the small intestine. During a developmental time course, we observed that FEnS form exclusively up to P2, whereas organoids are formed from P15 and onward (Figures 2D–2I). Interestingly, analysis of material from P2 to P15 illustrates the formation of both FEnS and organoids with an increasing fraction of the latter (Figures 2G−2I). Murine FEnS (mFEnS) are morphologically indistinguishable from hFEnS and can be expanded through fortnightly passaging for at least 2 years (Passage n ≈ 100). During their serial passaging we observe no spontaneous maturation or morphological and karyotypic alterations (Figure 2J). Although PGE2 is not required for maintenance of mFEnS, it does provide a pro-proliferative effect independent of Wnt signaling (Figure S2I). As has been reported for the adult colonic cultures, this is most likely via cAMP-mediated block of anoikis and stimulation of MAP kinase signaling (Jung et al., 2011). Established mFEnS can grow without R-spondin1 and in the presence of the natural Wnt antagonist DKK1, Porcupine inhibitor (which inhibits Wnt secretion), and tankyrase inhibitor (which stabilizes the Axin2/APC complex responsible for degradation of β-catenin), hereby demonstrating that FEnS can be maintained independently of Wnt signaling (Figures S2J and S2K). This distinguishes them from adult organoids.

Characterization of mFEnS revealed that they consist of a polarized epithelium with Villin localized to the apical surface, similar to the small intestine (Figures 2K and 2L). Moreover, FEnS phenocopy the differentiation patterns of the immature epithelium as there are no detectable secretory cell markers at both the protein and RNA level and reduced expression of adult stem cell markers (Figures 2M–2P and S3A). BrdU incorporation analysis showed that proliferative cells in mFEnS are scattered across the whole surface, whereas proliferative zones in organoids are restricted to the crypt domains (Figures 2Q and 2R). The overall morphology and growth of FEnS as spheres are reminiscent of that reported for organoids that form as a result of augmented Wnt signaling following loss of APC (Sato et al., 2011b). However, expression analysis demonstrates distinct expression patterns between FEnS and APCnull organoids (Figure S3B). In particular, it is clear that loss of APC causes increased levels of adult stem cell markers, whereas these are generally reduced in the fetal state (Figure S3B). In summary, this demonstrates that progenitors within the fetal small intestine have a unique behavior that sets them aside from both normal and cancerous adult stem cells.

### In Vitro Maturation of Fetal Enteric Progenitors

**Figure 3:**
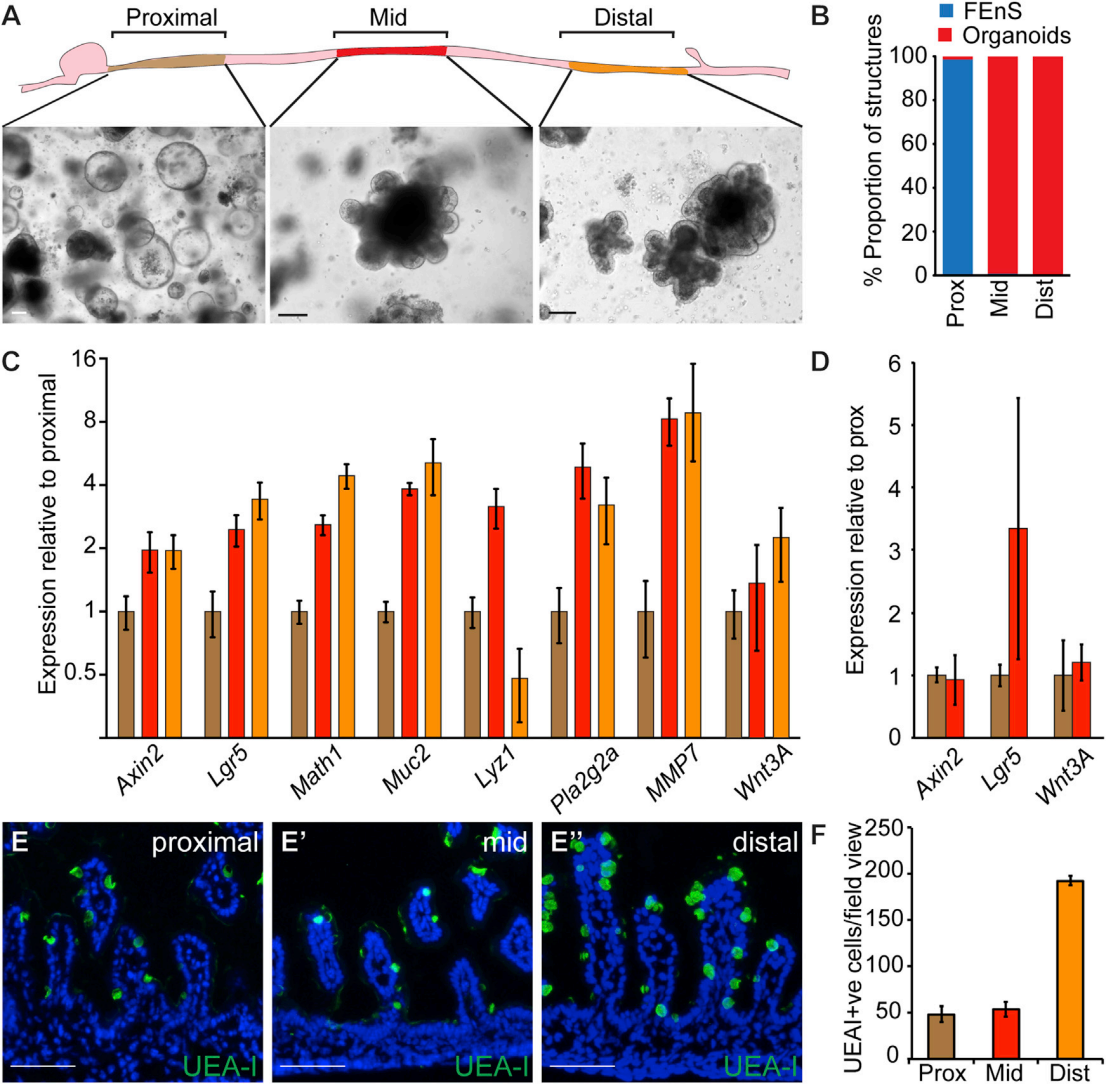
Adult Stem Cell Behavior Follows a Caudal to Rostral Pattern (A) Schematic diagram of the Proximal, Mid, and Distal parts of the small intestine and the representative images of cultures derived at P2. (B) Relative proportion of FEnS and organoids in the different sections of the small intestine. (C) Expression analysis in material isolated from Proximal, Mid, and Distal regions. Data represent the mean, and the error bars, the SEM (n = 3). Data are expressed relative to Proximal, on a Log_2_ scale. (D) Expression analysis of cultures from proximal and mid intestine enriched for FEnS and organoids, respectively. Data represent the mean, and the error bars, the SEM (n = 3), and are normalized to proximal cultures. (E) Detection of cells of the secretory lineage based on binding of *Ulex europaeus* agglutinin I (UEA-I) in the proximal, mid, and distal small intestine. (F) Quantification of UEA-I^+ve^ cells. Data represent the mean, and the error bars, the SEM (n = 3). The scale bars represent 100 μm.

Intestinal maturation in vivo has been proposed to follow a wave from proximal to distal sites (Spence et al., 2011a). To assess the positional effect along the length of the small intestine, we analyzed the regional differences in in vitro growth potential at postnatal day 2 (Figure 3A). Contrary to expectations, FEnS formed from proximal tissue, whereas more distal tissues formed organoids (Figures 3A and 3B). Gene expression analysis showed that the ability to form organoids correlates with increased levels of *Lgr5* and *Axin2* (Figure 3C). Analysis of the cultured material from the proximal and mid regions of the small intestine shows variable but comparable expression of Wnt target genes, suggesting that FEnS can respond to Wnt stimulation and that this represents a transitory and dynamic cellular state (Figure 3D). In line with the observed adult stem cell behavior, the distal part of the small intestine expresses higher levels of secretory lineage markers, which are characteristic of the adult small intestine, and contains a greater number of *Ulex europaeus* agglutinin I (UEA-I) reactive secretory cells (Figures 3C, 3E–3E″, and 3F). This further supports a distal to proximal wave of tissue maturation.

**Figure 4:**
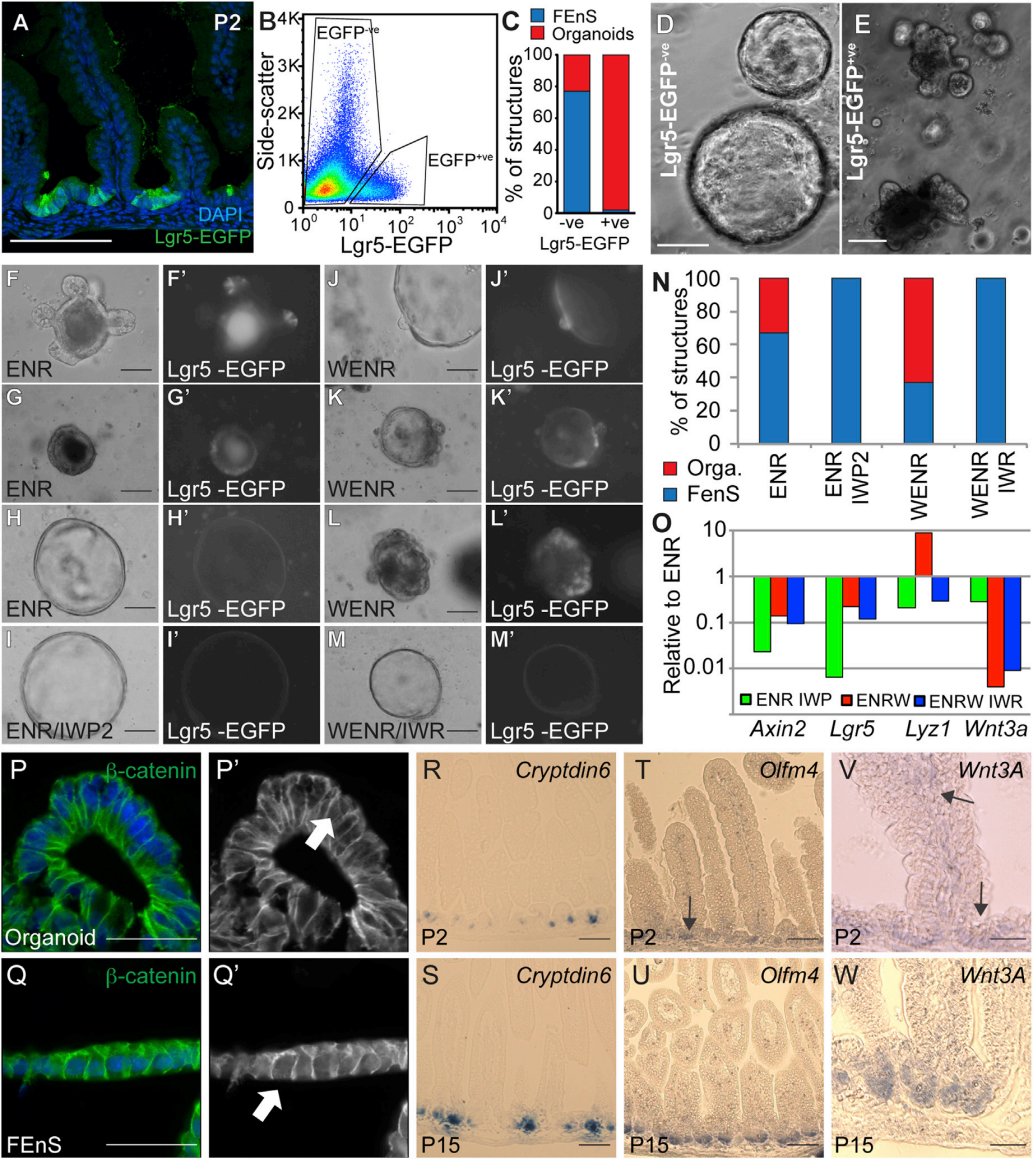
In Vitro Maturation of Fetal Enteric Progenitors Is Associated with Lgr5 Expression and Wnt Signaling (A) Detection of Lgr5-EGFP at P2 from Lgr5-EGFP-ires-CreERT2 mice. (B) Isolation of Lgr5-EGFP^+ve^ and Lgr5-EGFP^−ve^ epithelial cells from P2 small intestine by flow cytometry. (C) Quantification of proportion of FEnS and organoids formed in vitro from Lgr5-EGFP^−ve^ and Lgr5-EGFP^+ve^ neonatal intestinal epithelial cells. (D and E) Representative images of structures formed in vitro from Lgr5-EGFP^−ve^ and Lgr5-EGFP^+ve^ neonatal intestinal epithelial cells. (F–M) Representative images of FEnS and organoids derived from Lgr5-EGFP-ires-CreERT2 mice and cultured in the presence of EGF, Noggin, and R-spondin1 (ENR), ENR and the porcupine inhibitor IWP2 (ENR/IWP2), ENR and Wnt3a (WENR), or WENR in the presence of the tankyrase inhibitor IWR (WENR/IWR). (F′)–(M′) show grayscale images of EGFP in the derived structures. (N) Quantification of proportion of FEnS and organoids formed in the different treatment groups (ENR: 18/8; ENR/IWP2: 26/0; WENR: 21/30; WENR/IWR: 33/0). Two-tailed Fisher’s exact test shows significant difference between ENR and ENR/IWP2 (p = 0.0042), ENR and WENR (p = 0.0297), and WENR and WENR/IWR (p < 0.0001). (O) Expression analysis of the different treatment groups normalized to the ENR condition. Data represent the mean (n = 2). (P and Q) Detection of β-catenin (green) in organoids and FEnS. Arrows indicate cells with nuclear localization of β-catenin suggestive of active signaling. P‘-Q’ show β-catenin expression in grayscale. (R–W) In situ hybridization for *Cryptdin6*, *Olfm4*, and *Wnt3a* in tissue from P2 and P15. Arrows in (S) and (U) indicate regions of *Olfm4* and *Wnt3A* expression, respectively. The scale bars represent 50 μm (F, J, K–L, P–Q, and V–W) or 100 μm (A, D–E, G–I, M, and R–U). Cells are counterstained with DAPI (blue) in (A), (O), (P) and (Q). See also Figure S4 and Movie S1, Movie S2, and Movie S3.

In the mature intestine, Lgr5 marks ISCs, and single sorted Lgr5^+ve^ cells give rise to adult organoids (Barker et al., 2007; Sato et al., 2009). In the immature intestine Lgr5 is expressed by cells in the intervillus regions (Figure 4A). We hypothesized that Lgr5 expression defines progenitors permissive for transitioning into the adult state. In line with this, Lgr5-EGFP^+ve^ cells sorted from neonatal intestinal epithelium form organoids in vitro, whereas FEnS are formed from cells in the Lgr5-EGFP^−ve^ population (Figures 4B–4E). It is impossible to assess whether organoids form exclusively from Lgr5^+ve^ cells, as a large proportion of Lgr5-expressing cells in the Lgr5 knockin model are EGFP^−ve^ due to the mosaic nature of the mouse model.

To assess the relationship between organoids and FEnS, we analyzed samples from P2. Approximately one-half of the structures grow in a manner indistinguishable from fetal tissues (Figure S4A, Movie S1), whereas the rest followed a distinct pattern indicative of spontaneous differentiation (Figure S4B, Movie S2). All structures grow exponentially for around 7 days. At this point some structures collapse and start to form budding protrusions from the surface (Figure S4B). After passaging, these P2 organoids become R-spondin1 dependent and identical to structures obtained from more mature intestinal tissue (Figure S4C, Movie S3).

Since *Lgr5* and *Axin2* are both Wnt target genes, and given the dynamic regional expression correlating with organoid formation (Figure 3C), we investigated whether Wnt3a can induce intestinal maturation in vitro. Stimulation of cells from E16 proximal intestine, which normally only form FEnS, promoted the transition into budding organoids in a proportion of the forming structures (Figure S4D). This effect is enhanced upon passaging and the forming organoids can subsequently be maintained without exogenous Wnt in an R-spondin1-dependent manner (Figure S4Dix). Continued culture of organoids with high levels of exogenous Wnt3a produced the cystic morphology previously described for Wnt overactivity in adult cultures (Sato et al., 2011b; Figures S4Dviii). In contrast, FEnS could not be induced to transit to an adult state with Wnt3a (Figures S4Dvi and S4Dvii). The observed Wnt-stimulated maturation of FEnS to organoids is associated with the expected upregulation of secretory lineage markers (Figure S4E). It is clear that FEnS respond to Wnt stimulation, as *Lgr5* and *Axin2* expression is elevated compared to established cultures and also newly transitory organoids (Figure S4E); however, the signal is insufficient to induce maturation.

In order to further probe the functional significance of Wnt in the transition from a fetal to an adult phenotype, epithelial cells were isolated from P2 proximal small intestine. Because a proportion of FEnS at this stage naturally transition to the adult organoids, it is possible to investigate the importance of Wnt signaling in the establishment of both organoids and FEnS, as well as the transition between the two states. Epithelial cells were isolated from the Lgr5-reporter model in order to visualize Lgr5 expression. Whenever we observe high Lgr5-EGFP expression, this is in association with structures that are beginning to transition into the adult state. In medium supplemented with ENR, EGFP^+ve^ cells can be found either in mature crypt domains (Figures 4F and 4F′) or in regions with columnar morphology (Figures 4G and 4G′), whereas FEnS structures are seemingly EGFP^−ve^ or dim (Figures 4H and 4H′). The addition of Wnt increases the number of formed organoids (Figure 4N) and the regions of Lgr5 expression in the developing structures. This varies from single positive buds to extensive regions of Lgr5-EGFP^+ve^ cells (Figures 4J–4L′). The transition and Lgr5 expression is blocked by the addition of porcupine inhibitor to ENR-supplemented cultures (Figures 4I, 4I′, and 4N) and by the addition of the tankyrase inhibitor IWR-1 to cells cultured in the presence of ENR and Wnt (Figures 4M, 4M′, and 4N). Importantly, these inhibitors do not preclude the formation of FEnS. The maturation is reflected at the RNA level, where Wnt induces a robust increase in the Paneth cell marker *Lysozyme*. However, it is also clear that FEnS in early cultures express endogenous *Wnt3a*, which drives both *Axin2* and *Lgr5* expression within the fetal population of cells (Figure 4O). In line with the elevated expression of *Axin2* and *Lgr5*, β-catenin can be observed in the nucleus of cells in FEnS as well as in the formed organoids (Figures 4P, 4P′, 4Q and 4Q′).

In vivo tissue maturation correlates with the emergence of secretory Paneth cells, which have been identified as the major source of epithelial Wnt secretion in the intestinal epithelium (Sato et al., 2011b; Farin et al., 2012). Although mature Lysozyme^+ve^ Paneth cells cannot be observed until postnatal week 2 (Figure S2E–S2H), these are preceded by immature secretory cells, which can be detected based on *Cryptdin6* expression (Wong et al., 2012). Assessment of tissues from P2 and P15 demonstrates that *Cryptdin6*-expressing cells can be detected as early as P2 (Figures 4R and 4S). This correlates with the appearance of cells that are weakly positive for the stem cell marker *Olfm4* as well as *Wnt3a* within the bottom of the intervillus regions (Figures 4T–4W). This provides an epithelial source of Wnt3a that can drive tissue maturation.

In summary, this demonstrates that exogenous Wnt induces elevated focal Lgr5 upregulation in the fetal state and that maturation proceeds from these Lgr5 expression domains. Expression of *Wnt3a* can be detected in proliferative intervillus regions as the tissue proceeds into its adult state, suggesting that Wnt induction in vivo correlates with tissue maturation.

### Regeneration of Adult Colonic Epithelium from mFEnS

**Figure 5:**
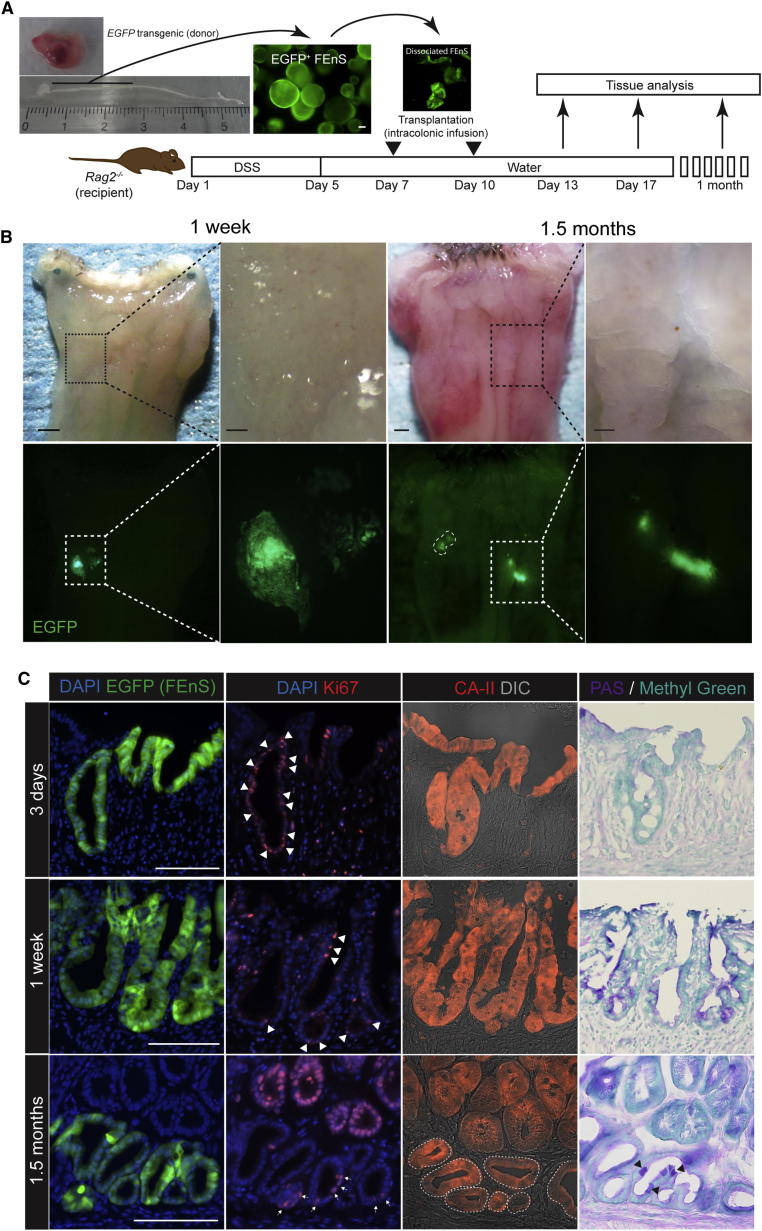
Regeneration of Adult Colonic Epithelium from mFEnS (A) Experimental protocol: gastrointestinal tract dissected from E16 EGFP transgenic mouse fetus (top left). Proximal small intestine was cultured in vitro as FEnS before mechanical dissociation and intracolonic transplantation into *Rag2*^*−/−*^ adult recipients with Dextran Sulfate Sodium (DSS)-induced ulcerative colitis. (B) Recipient colon at 1 week and 1.5 months posttransplantation. Lower panel shows EGFP^+ve^ areas in host colon. (C) Immunohistological analysis of EGFP^+ve^ fetal-derived engraftments for Ki67 (Ki67^+ve^ cells marked by arrowheads), carbonic anhydrase II, and PAS, 3 days, 1 week, and 1.5 months after transplantation. The scale bars represent 1 mm (whole colons) and 200 μm (magnified areas) in (B) and 100 μm in (C). See also Figure S5.

To assess the differentiation potential of immature intestinal progenitors and whether they represent a transplantable source, EGFP^+ve^ established mFEnS were injected under the renal capsule of mice (n = 8). In all cases at analysis, EGFP FEnS cells had either not proliferated or were not detectable. To test a more physiologically relevant approach, we transplanted EGFP FEnS into a chemically induced colonic injury model, where the repair process is associated with endogenous activation of Wnt signaling (Figure 5A; Yui et al., 2012; Koch et al., 2011). Within 3 hr after the first transplantation, FEnS-derived cells attached to ulcerated regions in the distal colon and were subsequently maintained long-term (Figures 5B and S5A–S5H). Initially, cells engrafted as a single-layered epithelium on top of the denuded lamina propria (Figures S5I and S5J). Three days following transplantation, grafted regions migrated downward into the underlying mesenchyme. Here they formed epithelial “pockets” with a central lumen and Ki67^+ve^ cells distributed along the length (Figures 5C, S5K, and S5L). One week after the second transplantation, engrafted cells formed epithelial crypt-like structures. These fetal-derived cells, although refractory to maturation in vitro, adapt to the colonic tissues, with subsets of cells differentiating appropriately into Mucin-2^+ve^ and PAS^+ve^ goblet cells and starting to express carbonic anhydrase-II, a specific marker of colonic tissue. None of this was detected in FEnS (Figures 5C, S5L, S5N, and S5P–S5R). Importantly, the grafted material did not express markers normally associated with the small intestine such as Lysozyme and alkaline-phosphatase (Figures S5S–S5T). Fetal-derived colonic crypts persisted at 1.5 months after transplantation, with continued evidence for proper differentiation and proliferation (Figures 5C, S5O, and S5P). Thus, immature enteric progenitors represent a transplantable source of cells with the capacity to differentiate in vivo.

## Discussion

In this study, we reveal the existence of a transitory population of progenitors present during the intestinal growth phase in both human and murine tissues. Moreover, a population of cells with similar characteristics can be obtained from pluripotent stem cells. This population is characterized by distinct proliferative and differentiation potential and reduced in vitro growth factor requirements compared to progenitors in the adult intestinal epithelium. Transition of fetal enteric progenitors into an adult state can be induced in vitro via stimulation with high levels of Wnt or alternatively by transplantation in vivo into an injury model. These cells are a valuable asset for understanding tissue maturation and an attractive source of transplantable progenitors for regenerative therapies.

Studies of human organ development are complicated by the availability of material. We provide evidence that mouse and human fetal intestine contain an immature population of epithelial progenitors and that similar immature cells can be obtained from hPSCs. Here the immature progenitors represent a transitory population of cells. Interestingly, many differentiation protocols from PSCs result in cells with a stable immature phenotype (Meyer et al., 2009; Nicholas et al., 2013). Based on our results this is not necessarily a tissue culture artifact but rather a result of the in vitro stabilization of an otherwise transitory state in vivo. It is however clear that it is not straightforward to extrapolate growth factor requirements from mouse to human cells as has been reported for their adult counterparts (Jung et al., 2011; Sato et al., 2009, 2011a).

Intestinal maturation has been proposed to follow a rostral-to-caudal (proximal-to-distal) wave (Spence et al., 2011a). We observe that FEnS form from the proximal region and organoids from the distal region, indicating that maturation in actual fact proceeds in the opposite direction. This is correlated with the expression pattern of markers of the mature secretory lineage and correlates with the observation that Lgr5 expression is associated with progenitors in a transitory competent state. These spatial and temporal observations are in agreement with previous work showing that Lgr5 gene expression is higher in the ileum than in the duodenum at E18.5 (Garcia et al., 2009). It does remain a possibility that the culture conditions that maintain adult stem cells in vitro are optimal for the distal intestine at this developmental time point rather than a reflection of tissue maturation.

The spatial differences in expression of the Wnt target genes *Lgr5* and *Axin2* (Barker et al., 2007; Lustig et al., 2002) prompted the investigation of Wnt signaling in the developmental transition. The differing requirements between the mature and immature states imply that Wnt signaling has a context-dependent role in development and tissue homeostasis or alternatively that ligands are dynamically regulated. There are several potential Wnt ligands in the intestine, where Wnt3a has been shown to play an autonomous role in epithelial stem cell maintenance (Sato et al., 2011b; Farin et al., 2012). In line with this, we observe that the expression of *Wnt3A* is correlated with the appearance of adult stem cell markers as well as adult stem cell behavior in the developing epithelium. Interestingly, this pattern of expression coincides with the phenotype of the knockout of the major β-catenin effector, Tcf4, which die shortly after birth with intestinal hypoplasia (Korinek et al., 1998).

In vitro Wnt stimulation and spontaneous maturation can be blocked by Wnt inhibition. Here, Wnt causes a prominent focal upregulation of Lgr5 expression in the developing structures. This is associated with the transition from a thin epithelium to domains with columnar morphology reminiscent of the cellular architecture in the small intestine. After the emergence of Paneth cells, these structures become independent of exogenous Wnt similar to adult intestinal stem cells. We hypothesize that Lgr5 in this context facilitates the transition by enhancing focal Wnt stimulation via the Wnt agonist R-spondin1 (de Lau et al., 2011). This will also explain why established FEnS are resilient to Wnt stimulation in vitro—they express significantly reduced levels of Lgr5. Although Wnt signaling mediates the transitioning of murine FEnS, it might be more complicated for hFEnS, where a low level of Wnt stimulation is required for their normal maintenance.

The gold standard for testing the true differentiation potential of progenitor cells is in vivo transplantation (Lin et al., 2013). We have previously demonstrated that adult colonic organoids can engraft into an injury model (Yui et al., 2012). An initial concern was that due to the striking morphological and growth similarities between FEnS and APC null adult organoids (Sato et al., 2011b), transplantation of FEnS in vivo would lead to tumor formation. However, FEnS cells were shown to attach to denuded regions of colonic epithelium and subsequently be incorporated into the colonic epithelium. Furthermore, since FEnS were unable to survive under the kidney capsule, this suggests that orthotopic transplantation is a more useful readout of in vivo potential. Our transplantation experiments unequivocally demonstrate that established FEnS can mature in vivo and contribute to regeneration of damaged gut epithelium in adult hosts. Moreover, it is striking that these fetal derivatives from the small intestine rapidly respond to the new microenvironment and differentiate appropriately to the regional requirements. This might reflect their immature behavior although we cannot exclude the possibility that adult organoids will behave similarly.

In summary, we have identified a population of expandable fetal enteric progenitors from mouse and human that can be used as a transplantable source. This work has important implications for understanding the mechanisms underlying intestinal maturation and demonstrates that immature intestinal progenitors, including fetal-like material derived from human pluripotent stem cells, have the potential to be used in colonic regenerative medicine. It will be interesting to see if similar populations of immature progenitors exist in other endodermal organs.

## Experimental Procedures

### Mice

*Rag2*^*−/−*^ mice were from Taconic Farms and Central Laboratories for Experimental Animals. *EGFP* transgenic mice and Lgr5-EGFP-ires-CreERT2 mice are described elsewhere (Barker et al., 2007; Okabe et al., 1997). Experimental animals were obtained by crossing these with C57BL/6 male or female animals. All animal experiments in Cambridge were performed under the terms of a UK Home Office License and transplantation experiments were performed with the approval of the Institutional Animal Care and Use committee of TMDU.

### Transplantation

Transplantation was performed as described on days 7 and 10 following initiation of dextran sulfate sodium-induced colonic injury (Yui et al., 2012). Donor FEnS were released from the Matrigel and mechanically dissociated into small sheets of epithelial tissue. Cell fragments from 500–1,000 FEnS were resuspended in 200 μl of Matrigel in PBS (1:20), which was instilled into the colonic lumen using a syringe and a thin flexible catheter. Animals were subsequently sacrificed at indicated time points.

### In Vitro Cultures

#### Organoids

Primary crypts from proximal adult small intestine were cultured as previously described with reduced concentration of murine recombinant R-spondin1 (500 ng/ml, R&D Systems; Sato et al., 2009).

#### FEnS

Fetal small intestines were opened longitudinally and cut into small pieces prior to dissociation with 2 mM EDTA. Isolated epithelial units were embedded in Matrigel and maintained in conditions identical to those used for adult organoids. In certain experiments Wnt3a and R-spondin1 from conditioned media were collected from HEK293 cell lines expressing recombinant Wnt3a and R-spondin1 (kindly provided by Hans Clevers and Calvin Kuo, respectively). Relative Wnt/R-spondin1 activity was measured using a TOPflash assay with a Dual-Luciferase Reporter Assay System (Millipore).

### Human Tissue

First-trimester human fetal material was obtained from the John van Geest Centre for Brain Repair, University of Cambridge, and used with informed consent under an Approved Protocol of Human Tissue Studies. Fetuses were staged by Crown Rump Length. Fetal intestines were processed for in vitro epithelial culture, paraffin sections, or RNA extraction, using procedures identical to those described for murine material, with the addition of PGE2 (2.5 μM, Sigma-Aldrich). Adult human intestinal biopsies were obtained from the Division of Gastroenterology and Hepatology, Department of Medicine, University of Cambridge, and were used with local ethical permission, under informed consent.

Adult primary human organoids were derived from biopsies obtained during routine colonoscopies from the terminal ileum. A single crypt suspension was obtained through chelation of the washed biopsies in cold chelation buffer (distilled water with 5.6 mmol/l Na_2_HPO_4_, 8.0 mmol/l KH_2_PO_4_, 96.2 mmol/l NaCl, 1.6 mmol/l KCl, 43.4 mmol/l sucrose, 54.9 mmol/l D-sorbitol, 0.5 mmol/l DL-dithiothreitol) containing 4 mM EDTA for 45 min followed by release of the crypts in fresh chelation buffer by vigorous shaking. Isolated crypts were treated like murine and fetal tissues; however, cultures were additionally supplemented with 1xN2 and 1xB27 (from invitrogen), 2.5 mM *N*-acetylcysteine (Sigma), 40% Wnt3a conditioned medium, 10% R-spondin1 conditioned medium, 10 mM nicotinamide, 10 μM SB202190, and 500 nM A-83-01. Tissue for primary human cultures was obtained at Herlev Hospital with local ethical permission and under informed consent.

### Generation, Culture, and Differentiation of hiPSCs

hiPSCs (BBHX8) were derived using retrovirus-mediated reprogramming of human skin fibroblasts (Rashid et al., 2010). hiPSCs were cultured in a chemically defined, feeder-free culture system (Brown et al., 2011). Cells were passaged every 7 days using a mixture of collagenase IV or collagenase and dispase at a ratio of 1:1. hiPSCs were differentiated as outlined in Figure S1 and Table S1 (Hannan et al., 2013). Briefly, iPSCs were differentiated into DE using Activin-A, BMP4, and LY294002 for 3 days. DE cells were subsequently cultured with CHIR99021 for 4 days to generate posterior endoderm. Raised aggregates of posteriorized endoderm were transferred into growth factor-reduced Matrigel. The cell-Matrigel mix was overlaid with Advanced DMEM/F12 supplemented with 2 mM GlutaMax (Invitrogen), 10 mM HEPES, and 100 U/ml Penicillin/100 mg/ml Streptomycin containing B27 supplement, Y-27632 (10 mM), human Noggin (100 ng/ml), human EGF (100 ng/ml), human R-spondin1 (1 mg/ml), and human Wnt3a (100 ng/ml).

### Imaging and Histology

Live imaging of 3D cultures was performed using a Nikon Biostation IM system. Structures in Matrigel were observed using phase contrast and DIC microscopy using an Axiovert 200M microscope (Zeiss) equipped with an AxioCam MRc (Zeiss).

Tissue preparation, staining, and image analysis were carried out as described previously using antibodies listed in Table S2 (Wong et al., 2012; Yui et al., 2012). Images of sections were acquired using a DeltaVision system (Applied Precision) or a Zeiss Imager M.2, equipped with AxioCam MRm and MRc cameras.

DIG in situ hybridization was carried out essentially as described before using IMAGE clones (Gregorieff et al., 2005).

### RNA Extraction and qRT-PCR

RNA was isolated from intact intestine as described (Wong et al., 2012). Total RNA was isolated from cultured cells using the Invitrogen PureLink RNA micro kit. cDNA was synthesized from 100 ng total RNA using the Invitrogen SuperScript III Reverse transcriptase kit, using random primers. Gene-specific expression assays (Applied Biosystems) or SYBR Green analysis (Invitrogen) with optimized primer pairs was used for qPCR on an Applied Biosystems 7500HT RealTime PCR System (Applied Biosystems). Values were normalized to 18S using the ΔCt method. Z scores were calculated and used to generate heatmaps in R.

### Isolation of Cells for Flow Cytometry

Cells were isolated essentially as described (Wong et al., 2012). A single-cell suspension was achieved by subsequent incubation using trypsin. Cell sorting was carried out using a MoFlo (Beckman Coulter). Ten thousand cells were seeded into 25 μl Matrigel. Data analysis was performed in FlowJo.

### Statistical Analysis

Statistical significance of quantitative data was determined by applying a two-tailed Student’s t test to raw values or to the average values obtained from analysis of independent experiments. A two-tailed Fisher’s exact test was used to analyze the significance of the Wnt and inhibitor culture experiment.

## Author Contributions

R.P.F., S.Y., and K.B.J. conceived and designed the study, analyzed the data, and wrote the manuscript; R.P.F., S.Y., N.R.F.H., C.S., A.M., P.J.S., and K.B.J performed experimental work; and R.P.F., S.Y., and K.B.J. prepared the figures. O.H.N., L.V., R.A.P., and M.W. gave conceptual advice. T.N. and K.B.J supervised the project.

## Supplemental Information

Document S1. Figures S1–S5 and Tables S1 and S2

Movie S1. Live Imaging of P2 Intestinal Epithelium Growing as a Fetal Enterosphere in 3D Culture Imaged over the Course of 10 Days (Related to Figure 4)

Movie S2. Live Imaging of P2 Intestinal Epithelium Maturing in 3D Culture Imaged over the Course of 10 Days (Related to Figure 4)

Movie S3. Live Imaging of Organoid Formation in 3D Culture from Adult Intestinal Epithelium Imaged over the Course of 8 Days (Related to Figure 4)
