# Transplantation of Expanded Fetal Intestinal Progenitors Contributes to Colon Regeneration after Injury

**DOI:** 10.1016/j.stem.2026.10.008

**Published:** 2026-11-05

**Authors:** Robert P. Fordham, Shiro Yui, Nicholas R.F. Hannan, Christoffer Soendergaard, Alison Madgwick, Pawel J. Schweiger, Ole H. Nielsen, Ludovic Vallier, Roger A. Pedersen, Tetsuya Nakamura, Mamoru Watanabe, Kim B. Jensen

## Main text

(Cell Stem Cell *13*, 734–744; December 5, 2013)

In the originally published version of this article, the BrdU immunofluorescence image shown in Figure 2Q was inadvertently duplicated from an earlier publication from the authors’ laboratory. Following a detailed review of their archived data, the authors determined that the mistake originated during figure assembly. Specifically, an image from the 2012 study had been saved under an incorrect file name, which led to its accidental inclusion in the 2013 manuscript. This correction does not alter the interpretation of the experiment, or any of the conclusions of the paper. The authors take full responsibility for this oversight and regret that it was not identified prior to publication.

**Figure 2:**
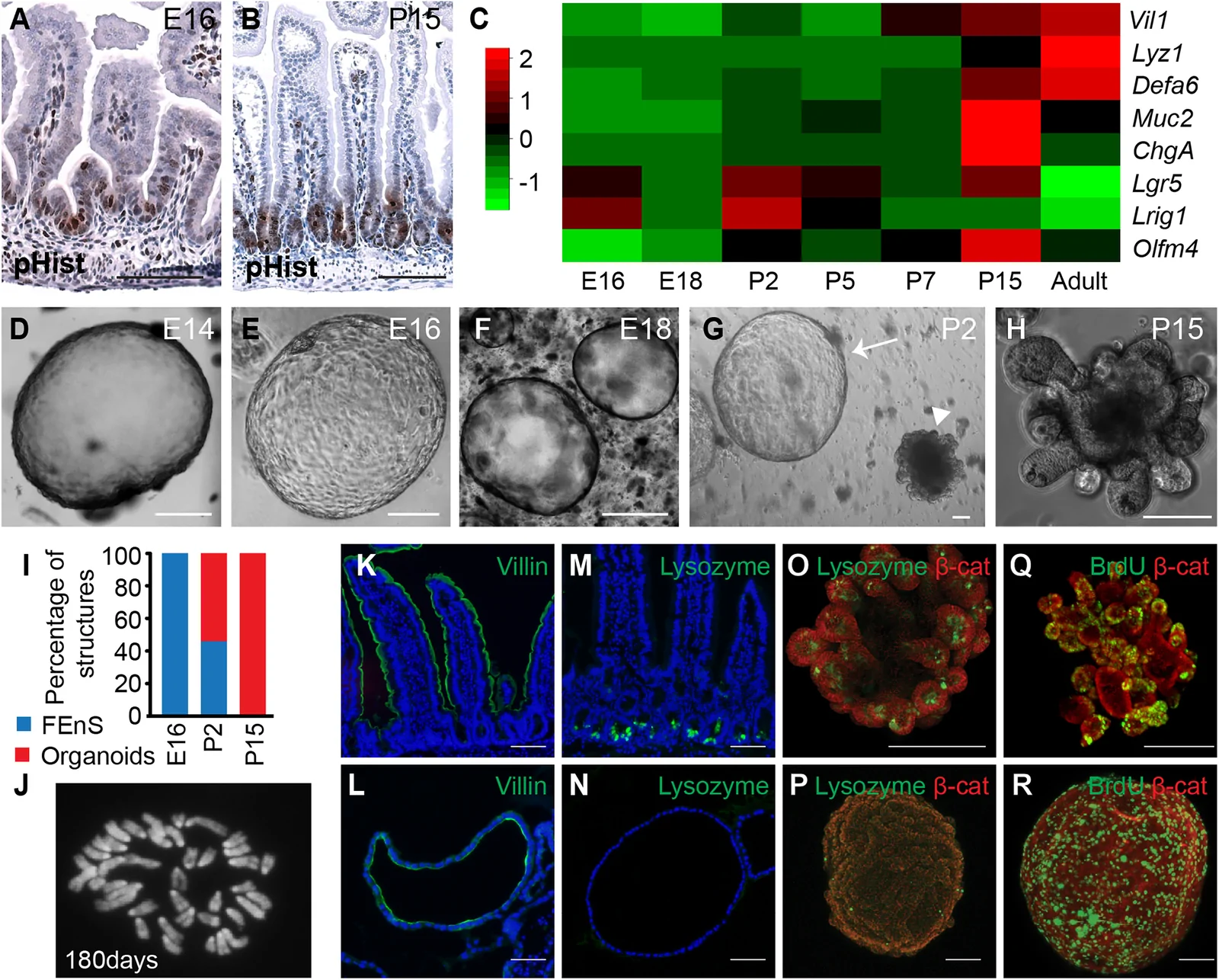
Establishment of mFEnS from Immature Mouse Intestine (corrected)

