# Supplementary material for: Transplantation of Expanded Fetal Intestinal Progenitors Contributes to Colon Regeneration after Injury
Source: Cell Stem Cell. 2013 Dec 5;13(6):734–44. doi: 10.1016/j.stem.2013.09.015 (PMC3858813; doi:10.1016/j.stem.2013.09.015)
Supplement: Document S1. Figures S1–S5 and Tables S1 and S2 [file mmc1.pdf]

Cell Stem Cell, volume *13*

Supplemental Information

Transplantation of Expanded Fetal Intestinal Progenitors Contributes to Colon Regeneration after Injury

Robert P. Fordham, Shiro Yui, Nicholas R. F. Hannan, Christoffer Søndergaard, Alison Madgwick, Pawel J. Schweiger, Ole H. Nielsen, Ludovic Vallier, Roger A. Pedersen, Tetsuya Nakamura, Mamoru Watanabe, Kim B. Jensen

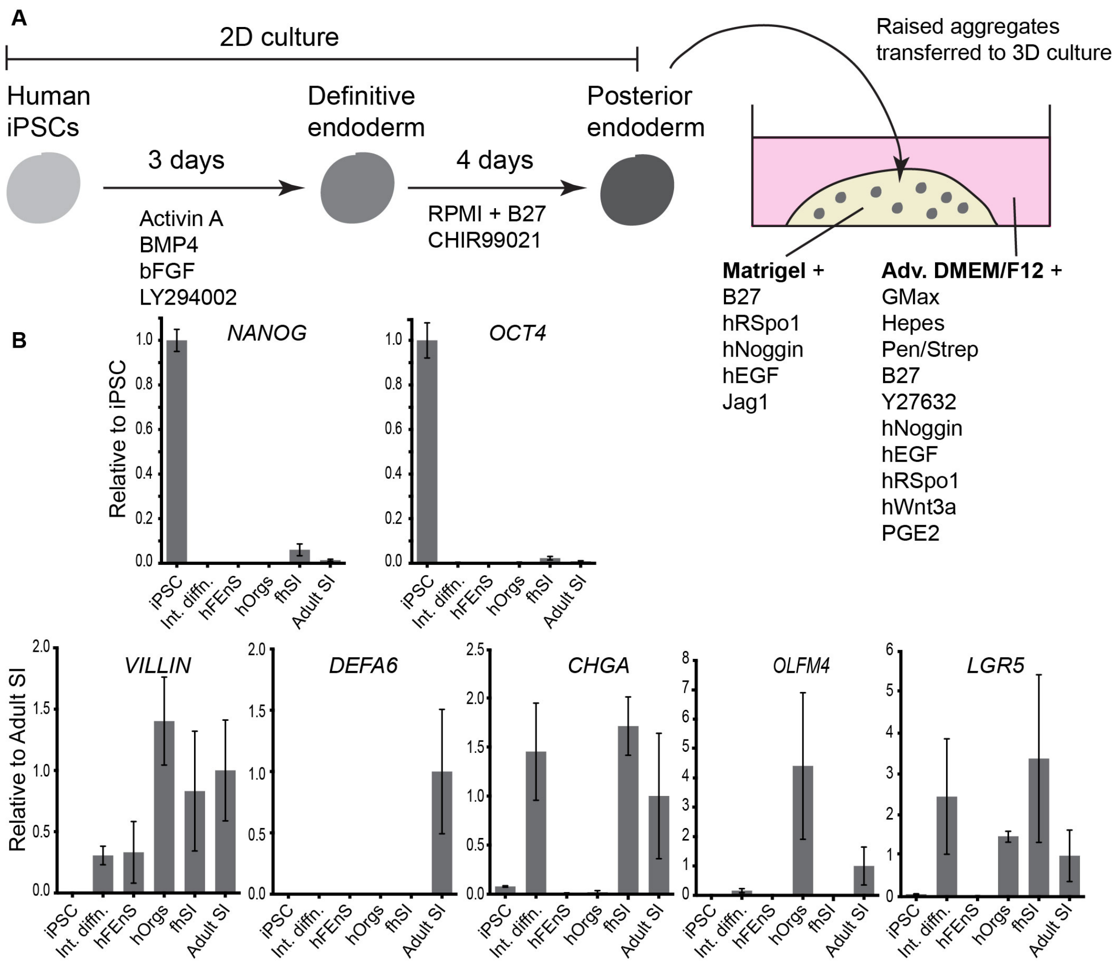

Figure S1 related to Figure 1. Differentiation of human induced pluripotent stem cells to intestinal epithelium.

(A) Schematic protocol for intestinal differentiation of hPSCs. Also see Supplemental Table 1. **(B)** Relative expression levels of intestinal lineage markers in material from undifferentiated human induced pluripotent stem cells (hiPSC), iPSC-derived intestine (Int. diff.), primary fetal human enterospheres (hFEnS), human adult organoids (hOrgs), primary human fetal intestine (FhSI) and primary human adult intestine (AhSI). Data represent the mean and the error bars the SEM (n=3).

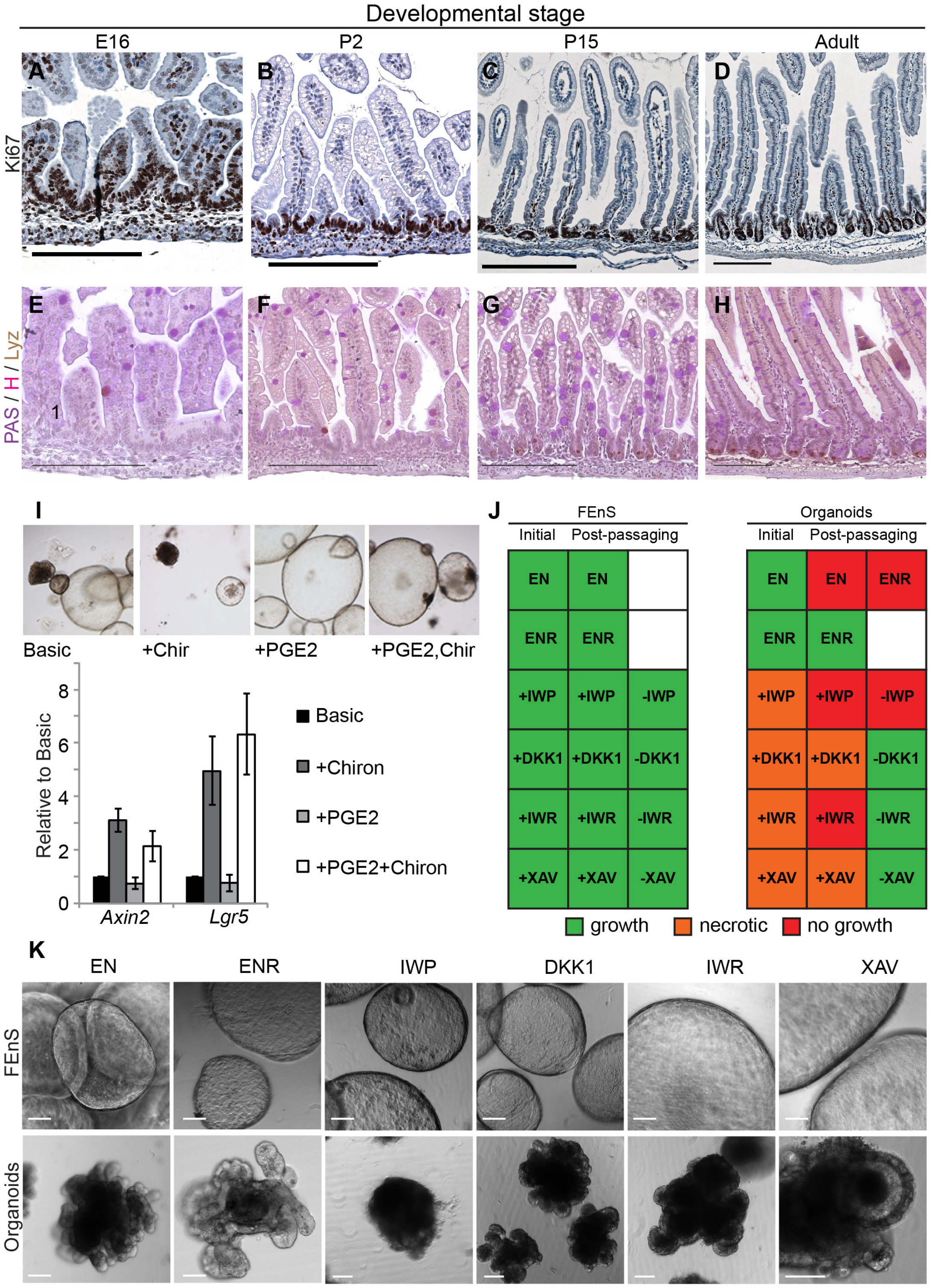

Figure S2, related to Figure 2. Development of the murine small intestine and characterization of growth factor requirements of FEnS.

(A-E) Immunohistochemistry for Ki67 nuclear antigen. **(E-H)** Immunohistochemistry for Lysozyme (Lyz) (brown), counterstained with Periodic Acid – Schiff's reagent (PAS) (purple) and haematoxylin (pink). **(I)** Expression analysis of mFEnS treated

with prostaglandin E2 (PGE2) and the GSK3 inhibitor Chiron (Chir) in the presence of EGF, Noggin and R-spondin (Basic). Data are expressed relative to ENR conditions and represent the mean and the error bars SEM (n=3). **(J-K)** Assessment of the role of Wnt signaling in maintenance of FEnS. Table is color coded according to the behavior of the treated FEnS both during primary treatment, following passaging and subsequent culture in the presence and absence of inhibitor. EN (EGF+Noggin), and ENR supplemented with either IWP (Porcupine inhibitor), DKK1 (Dickkopf1), IWR and XAV (Tankyrase inhibitors). The scale bars represent 100µm (K) and 200µm (A-H).

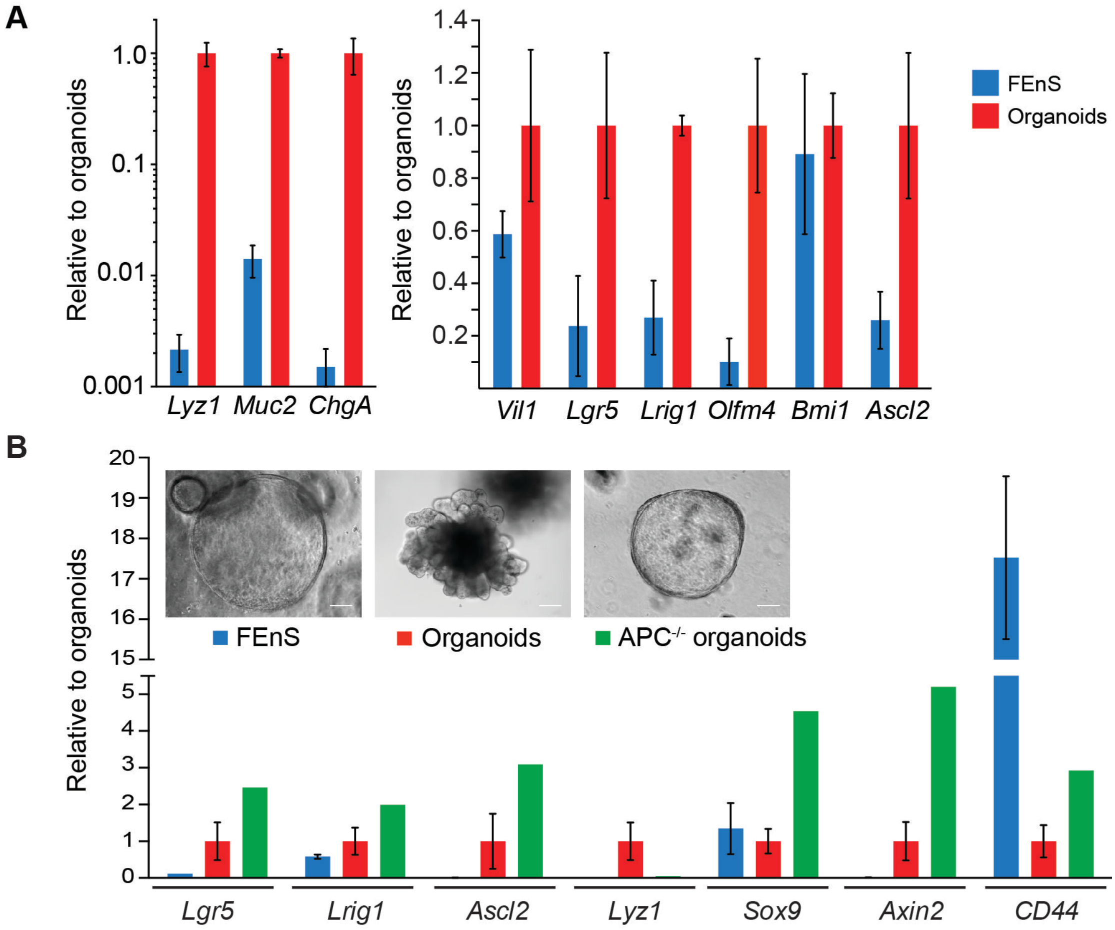

Figure S3, related to Figure 2: Charaterization of mFEnS with respect to their molecular phenotype.

(A) Expression analysis for markers of secretory lineages, absorptive cells and stem cells in mouse FEnS and organoids. Data are expressed relative to organoids and represent the mean and the error bars SEM (n=3). **(B)** Expression analysis of characterized Wnt responsive stem cell genes in FEnS, WT organoids and organoids derived from APC$^{-/-}$ tissue. Data are expressed relative to organoids and represent the mean and the error bars SEM (n=3, organoids and FEnS; n=1 for APC -/- organoids).

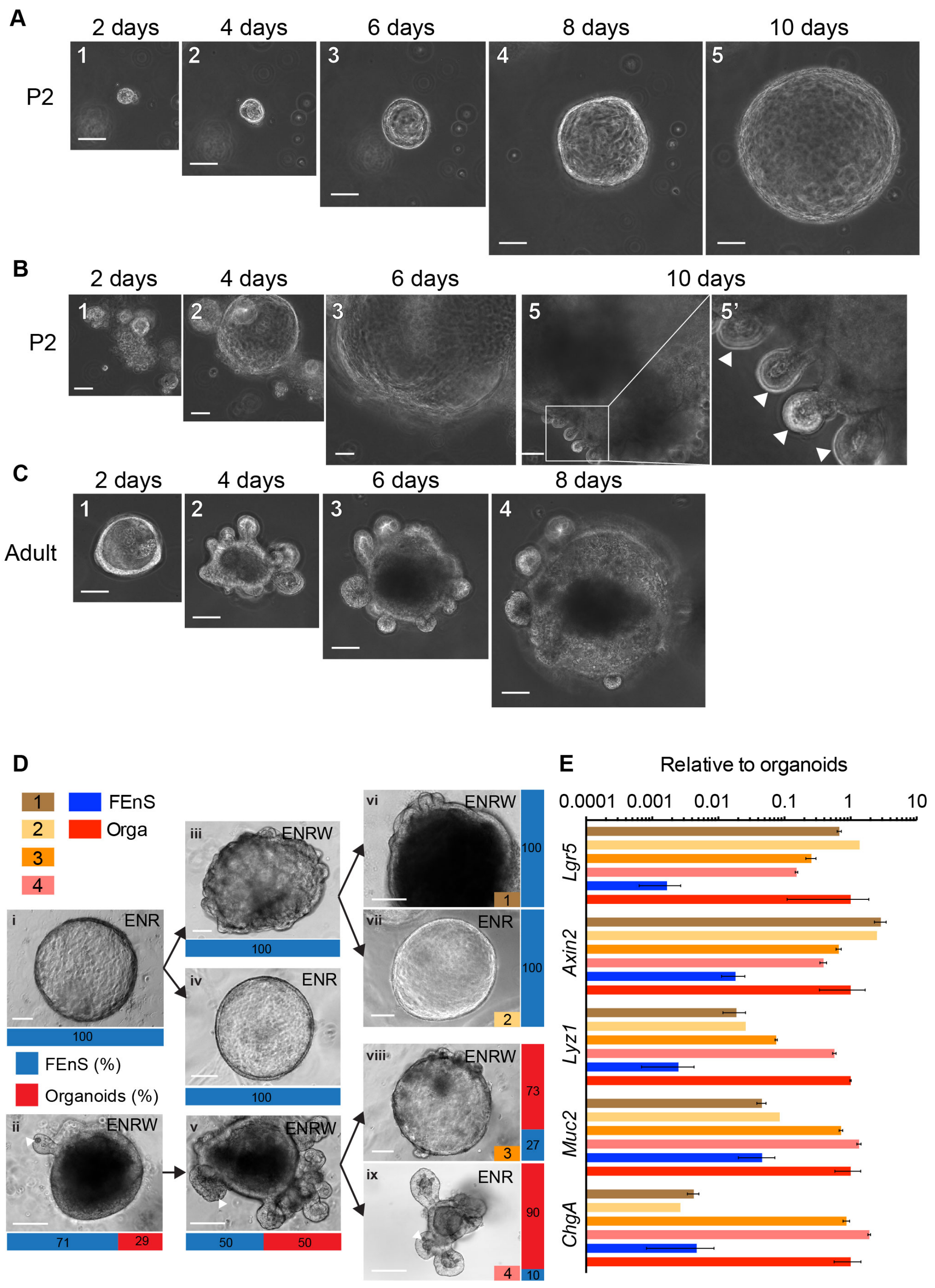

Figure S4, related to Figure 4. In vitro maturation of mFEnS.

(A-B) A non-budding FEnS structure and a spontaneously maturing structure from postnatal day two intestinal epithelium growing in three-dimensional *in vitro* culture, imaged over a period of ten days (Supplemental Movies 1 and 2). Arrow heads in 5' indicate mature crypts. **(C)** Growth of an intestinal epithelial organoid derived from

adult tissue, imaged over a period of eight days (Supplemental Movie 3). **(D)** Representative images of structures growing *in vitro* from E16 intestinal epithelium under different conditions: E, EGF; N, Noggin; R, R-Spondin-1; W, Wnt3a. Bar charts (underneath i-v, and alongside vi-ix) represent quantification of the relative proportion of FEnS and organoids (orga) found in each culture condition. n-numbers: (i) 310, (ii) 35, (iii) 8, (iv) 16, (v) 26, (vi) 40, (vii) 13, (viii) 71, (ix) 132. **(E)** Expression analysis for markers of secretory lineages and stem cells in cultures from the four treatment groups as well as established FEnS and organoids. Data are expressed relative to organoids and represent the mean and the error bars SEM (n=3). The scale bars represent 100 μm.

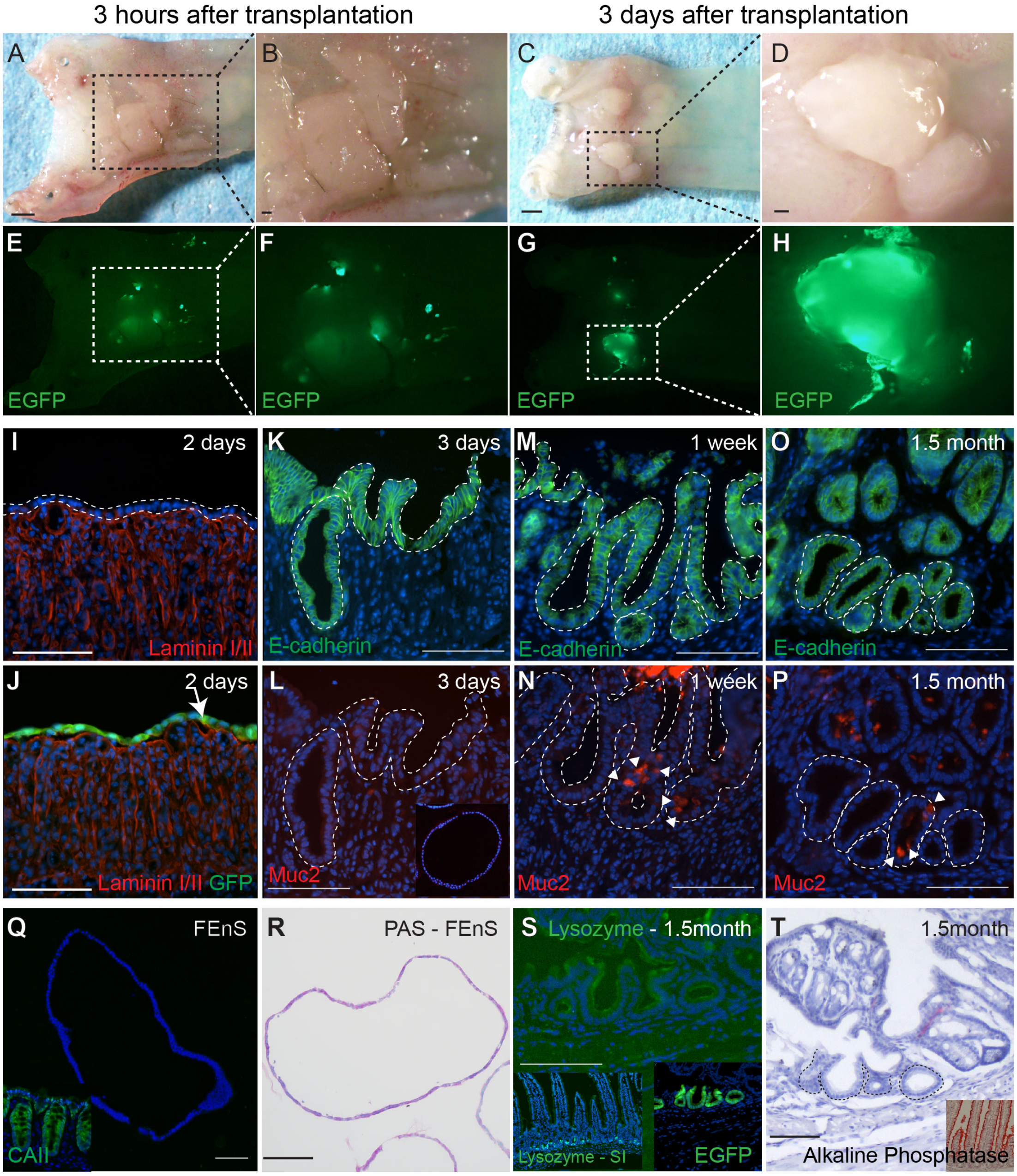

Figure S5, related to Figure 5. FEnS engraftment and crypt formation *in vivo*

(A-D) FEnS engraftments (EGFP^{+ve} regions in E-H) in distal colon three hours (A,B,E,F) and three days (C,D,G,H) after transplantation. **(I, J)** FEnS-derived cells form an epithelial layer on top of the denuded Laminin I/II^{+ve} lamina propria, indistinguishable from host-derived Wound Associated Epithelium, shown between demarcated lines in (I). **(K-P)** Immunofluorescent staining of fetal-derived, colonic engraftments for E-Cadherin (K,M,O) and Mucin-2 (L,N,P) at 3 days (K,L), 1 week (M-N) and 1.5 months (O-P) after transplantation. Dashed outlines indicate EGFP^{+ve}

FEnS-derived crypts. The corresponding EGFP expression can be seen in Figure 5. Arrowheads indicate fetal-derived Mucin-2^{+ve} goblet cells within new crypts. Insert in (L) shows Mucin2 expression in FEnS. **(Q)** FEnS do not express carbonic anhydrase II (CAII). Insert shows positive control from colon. **(R)** FEnS used for the graft are PAS negative. **(S)** The graft is negative for Lysozyme expression. Insert bottom left is a positive control from the small intestine and bottom right shows EGFP expression in the grafted region. **(T)** Alkaline phosphatase cannot be detected in the grafted material, but is highly expressed in the small intestine (Insert). Dashed outline show the grafted region from R. Scale bars represent 1 mm (A,C), 200 μm (B,D), 100 μm (I-T).

Movie S1, related to Figure 4:

Live imaging of P2 intestinal epithelium growing as a Fetal Enterosphere (FEnS) in 3D culture imaged over the course of 10 days.

Movie S2, related to Figure 4:

Live imaging of P2 intestinal epithelium maturing in 3D culture imaged over the course of 10 days.

Movie S3, related to Figure 4:

Live imaging of organoid formation in 3D culture from adult intestinal epithelium imaged over the course of 8 days.

Table S1, related to Figure 1: Cell culture reagents. Chemical compounds, growth factors and medium utilized for the directed differentiation of human pluripotent stem cells to intestinal epithelium with the indicated concentrations and suppliers used.

**Component**	**Supplier**	**Concentration**
Activin-A	R & D Systems	100 ng/ml
BMP4	R & D Systems	10 ng/ml
LY294002	Promega	10 μM
RPMI 1640	Gibco	
B27 supplement	Gibco	
CHIR99021	StemGent	6 μM
Matrigel, Growth factor reduced	BD Biosciences	Undiluted
B27 (retinoic acid depleted)	Invitrogen	
R-spondin (human)	R & D Systems	500 ng/ml (in Matrigel); 1 μg/ml (in medium)
Noggin (human)	R & D Systems	100 ng/ml
EGF (human)	R & D Systems	100 ng/ml
Jagged-1	AnaSpec Inc.	1 μM
Advanced DMEM/F12	Gibco	
GlutaMax	Invitrogen	2 mM
Hepes	Invitrogen	10 mM
Penicillin	Gibco	100 U/ml
Streptomycin	Gibco	100 μg/ml
Y-27632	Sigma Aldrich	10 μM
Wnt3a (human)	R & D Systems	100 ng/ml
Prostaglandin-E2	Sigma Aldrich	2.5 μM
bFGF	R & D Systems	20 ng/ml

Table S2, related to Figures 1-5: List of antibodies. Summary of the antibodies used throughout the study with indicated clone name and species as well as the dilution used in the study for imaging and flow cytometry.

Reactive against	Raised in	Supplier and code	Dilution
β-catenin	Rabbit	Santa Cruz, sc-7199	1:1000
Phospho-histone H3 (Ser 10)	Rabbit	Cell Signaling, 9701	1:200
Ki67	Rat	Dako Cytomation, clone TEC3	1:50
Ki67	Mouse	Monosan, clone MM1	1:1000
Lysozyme	Mouse	Serotec, clone SB1 (BGN/06/961)	1:200
Lysozyme	Rabbit	Dako, EC 3.2.1.17	1:2000
Laminin I/II	Rabbit	Abcam, ab7463	1:500
BrdU	Mouse	Cell Signaling, Bu20a	1:250
Mucin-2	Rabbit	Santa Cruz, sc-15334	1:200
Carbonic anhydrase II (CAII)	Rabbit	Santa Cruz, sc-25596	1:500
E-cadherin	Mouse	BD Transduction, 610181	1:200
CD45 (PE-Cy7 conjugated)	Rat	BD Biosciences, 30-F11	1μl / 10^6 cells
CD31 (PE-Cy7 conjugated)	Rat	eBioscience, 390	1μl / 10^6 cells
EpCAM (APC conjugated)	Rat	eBioscience, G8.8	1μl / 10^6 cells
UEA-I-Atto488 conjugate		Sigma	1:500
