# Supplementary figures and images for: Transplantation of Expanded Fetal Intestinal Progenitors Contributes to Colon Regeneration after Injury
Source: Cell Stem Cell. 2013 Dec 5;13(6):734–44. doi: 10.1016/j.stem.2013.09.015 (PMC3858813; doi:10.1016/j.stem.2013.09.015)

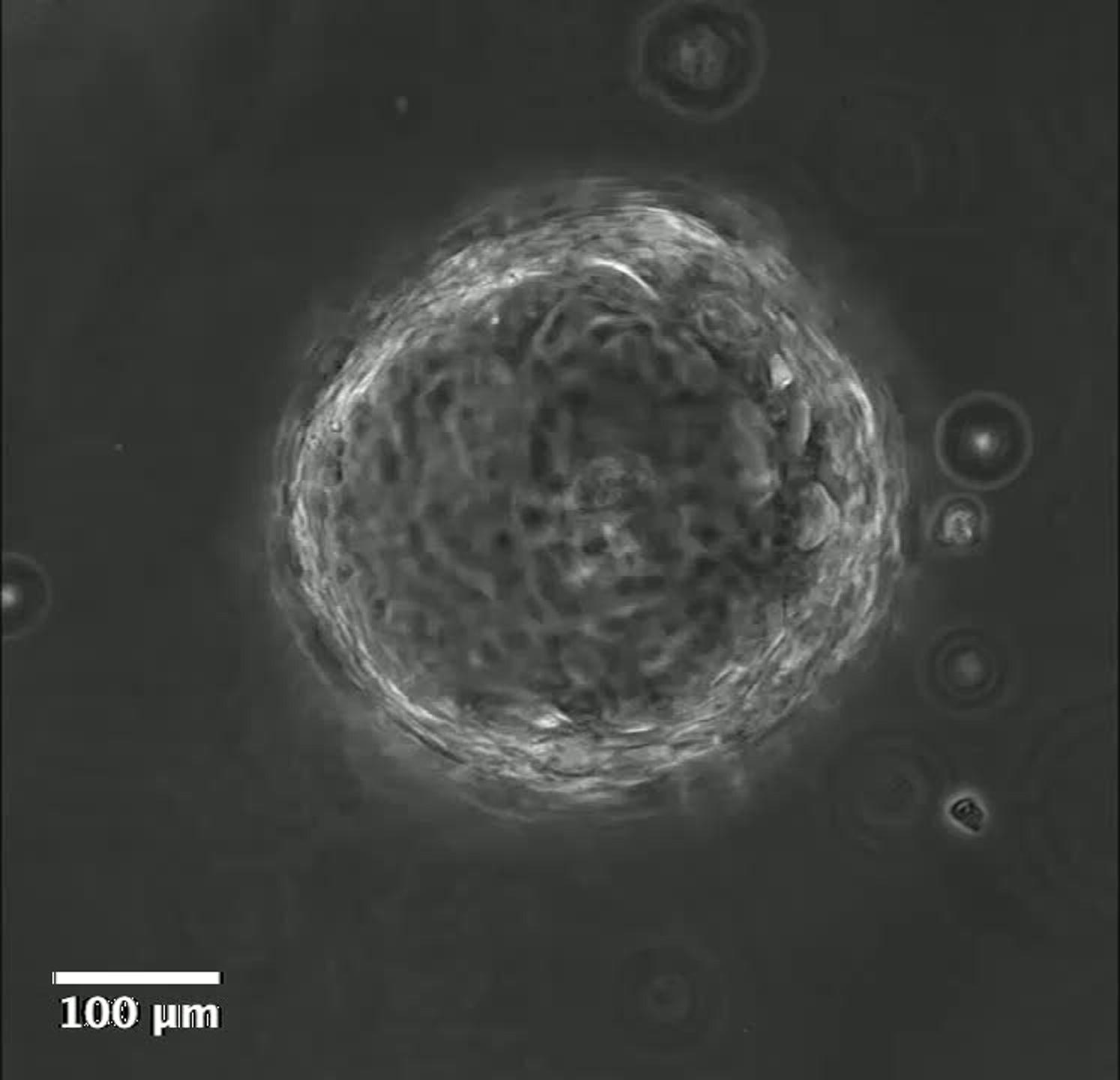

Supplement: Movie S1. Live Imaging of P2 Intestinal Epithelium Growing as a Fetal Enterosphere in 3D Culture Imaged over the Course of 10 Days (Related to Figure 4) [file mmc2.jpg]

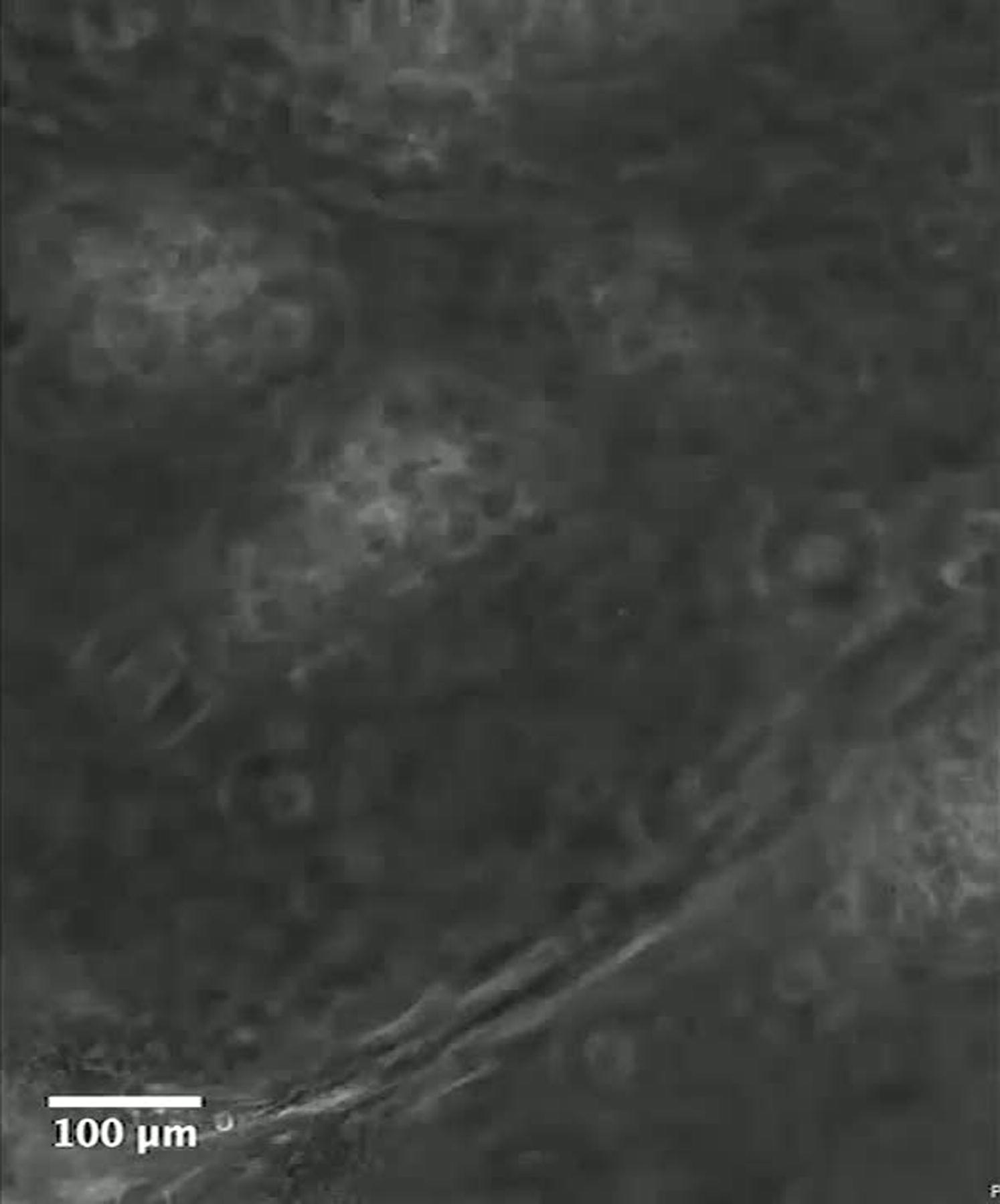

Supplement: Movie S2. Live Imaging of P2 Intestinal Epithelium Maturing in 3D Culture Imaged over the Course of 10 Days (Related to Figure 4) [file mmc3.jpg]

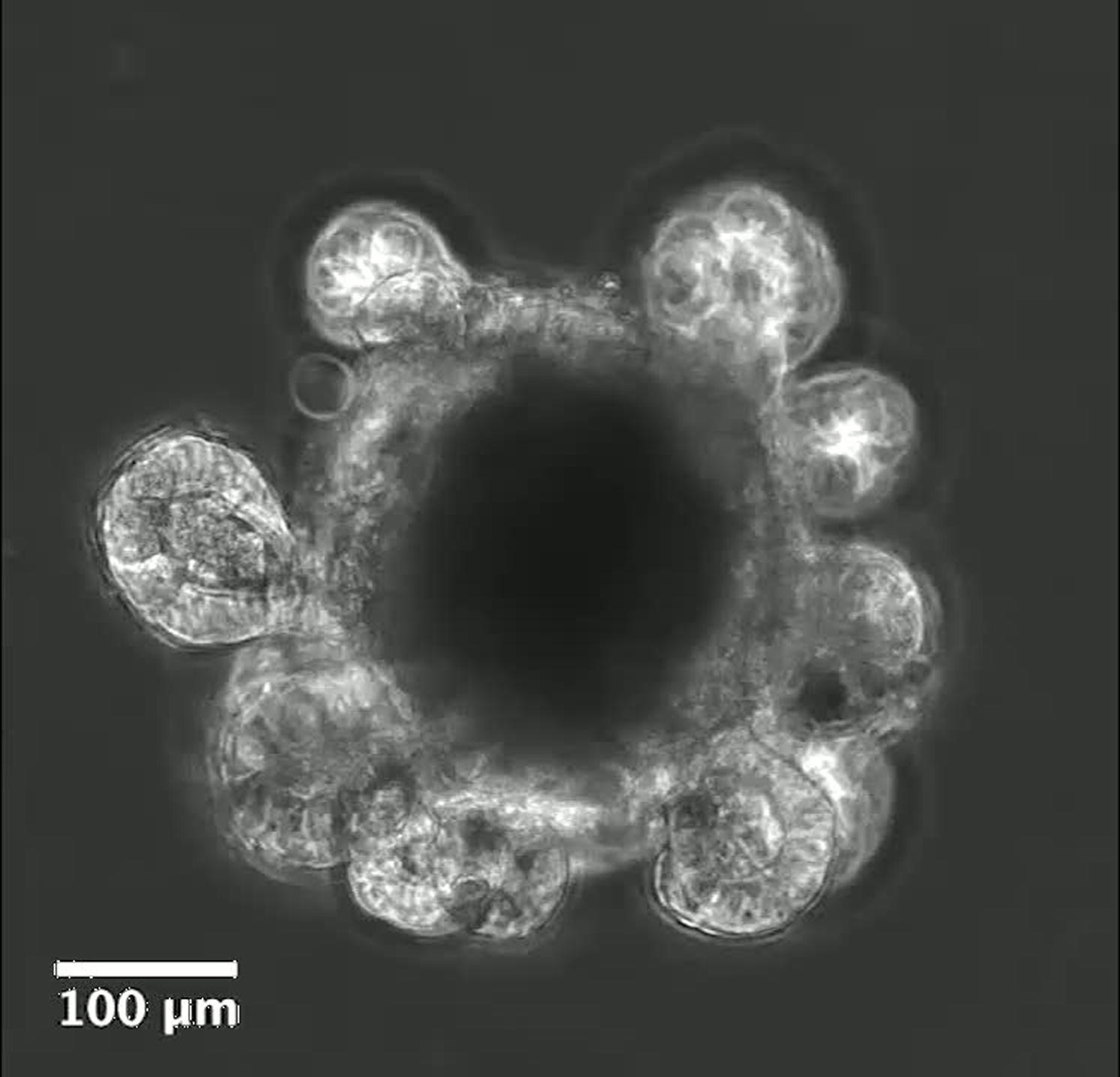

Supplement: Movie S3. Live Imaging of Organoid Formation in 3D Culture from Adult Intestinal Epithelium Imaged over the Course of 8 Days (Related to Figure 4) [file mmc4.jpg]
